# Water heater temperature set point and water use patterns influence *Legionella pneumophila* and associated microorganisms at the tap

**DOI:** 10.1186/s40168-015-0134-1

**Published:** 2015-12-01

**Authors:** William J. Rhoads, Pan Ji, Amy Pruden, Marc A. Edwards

**Affiliations:** Charles E. Via Department of Civil and Environmental Engineering, Virginia Tech, Blacksburg, VA 24061 USA

**Keywords:** *Legionella pneumophila*, Hot water, Stagnation, Water use, Temperature

## Abstract

**Background:**

Lowering water heater temperature set points and using less drinking water are common approaches to conserving water and energy; yet, there are discrepancies in past literature regarding the effects of water heater temperature and water use patterns on the occurrence of opportunistic pathogens, in particular *Legionella pneumophila*. Our objective was to conduct a controlled, replicated pilot-scale investigation to address this knowledge gap using continuously recirculating water heaters to examine five water heater set points (39–58 °C) under three water use conditions. We hypothesized that *L. pneumophila* levels at the tap depend on the collective influence of water heater temperature, flow frequency, and the resident plumbing ecology.

**Results:**

We confirmed temperature setting to be a critical factor in suppressing *L. pneumophila* growth both in continuously recirculating hot water lines and at distal taps. For example, at 51 °C, planktonic *L. pneumophila* in recirculating lines was reduced by a factor of 28.7 compared to 39 °C and was prevented from re-colonizing biofilm. However, *L. pneumophila* still persisted up to 58 °C, with evidence that it was growing under the conditions of this study. Further, exposure to 51 °C water in a low-use tap appeared to optimally select for *L. pneumophila* (e.g., 125 times greater numbers than in high-use taps). We subsequently explored relationships among *L. pneumophila* and other ecologically relevant microbes, noting that elevated temperature did not have a general disinfecting effect in terms of total bacterial numbers. We documented the relationship between *L. pneumophila* and *Legionella* spp., and noted several instances of correlations with *Vermamoeba vermiformis*, and generally found that there is a dynamic relationship with this amoeba host over the range of temperatures and water use frequencies examined.

**Conclusions:**

Our study provides a new window of understanding into the microbial ecology of potable hot water systems and helps to resolve past discrepancies in the literature regarding the influence of water temperature and stagnation on *L. pneumophila*, which is the cause of a growing number of outbreaks. This work is especially timely, given society’s movement towards “green” buildings and the need to reconcile innovations in building design with public health.

**Electronic supplementary material:**

The online version of this article (doi:10.1186/s40168-015-0134-1) contains supplementary material, which is available to authorized users.

## Background

The growth of opportunistic pathogens (OPs) in building plumbing systems is an increasing public health threat with no clear solutions [1–4]. In particular, the warm, stagnant conditions in building plumbing create ideal conditions for re-growth of a number of OPs and their free-living amoeba hosts (Additional file 1: Figure S1). *Legionella* spp., including *L. pneumophila*, are model organisms for understanding the interplay between building plumbing design and operation and OP proliferation. *Legionella* is now recognized as the most common agents of waterborne disease outbreak, resulting in an estimated 8000 to 18,000 hospitalizations (which are likely underreported) due to the severe pneumonia that characterizes Legionnaires’ disease [5, 6]. With community-acquired infections representing 96 % (*n* = 31) of reported drinking water-associated Legionnaires’ disease outbreaks from 2007 to 2010, the majority of drinking water-associated Legionnaires’ disease cases result from exposure to aerosols from drinking water systems in the built environment. A fundamental feature of *Legionella* and other OPs is that they can grow and thrive as part of complex microbial communities inhabiting building plumbing supplied by “clean” drinking water and therefore do not necessarily respond to traditional approaches for pathogen control geared towards fecal organisms [7, 8]. At the same time, the characteristic conditions in building plumbing (e.g., warm temperature, high surface area, and long residence time) make it difficult to maintain an effective chlorine residual usually depended upon to kill pathogens [3, 9, 10]. Thus, new strategies are needed for building plumbing design and operation that are informed by how they influence *Legionella* and its microbial ecology.

Prior field studies provide some clues about key factors that trigger *Legionella* colonization and amplification in building plumbing [11–13]. *Legionella* is notorious for growing in hot water systems, and while the optimal temperatures for inhibiting its growth have been well characterized in laboratory culture, they are not necessarily applicable to field conditions where *Legionella* colonizes biofilm and may be protected within an amoeba host [14]. Further, water heater set points do not directly translate into the temperature experienced at the tap, where it can quickly cool to room temperature.

Stagnation has also received a great deal of attention as a major risk factor for *Legionella* amplification and is interrelated with temperature setting [15–23]. For example, an advantage of recirculating systems is that they maintain water temperature in the recirculating pipes closer to the water heater temperature, which will ideally kill *Legionella* and prevent further colonization. Although the majority of guidance criteria advise against stagnation [17–19, 22], prior reports are inconsistent and indicate that it sometimes stimulates [15] and sometimes deters [23–25] *Legionella* growth. In the absence of disinfectant (thermal or chemical), stagnation may limit the delivery of new nutrients to distal taps, reducing the potential for re-growth [23, 26]. However, nutrient gradients have not been examined in an integrated fashion considerate of how plumbing temperature and water use conditions together might ultimately impact nutrient availability. Finally, given that *Legionella* and many other OPs are intimately dependent on host free-living amoebae for their replication in oligotrophic drinking water systems, better understanding this ecological relationship as influenced by temperature and stagnation is critical [14, 27, 28].

**Fig. 1:**
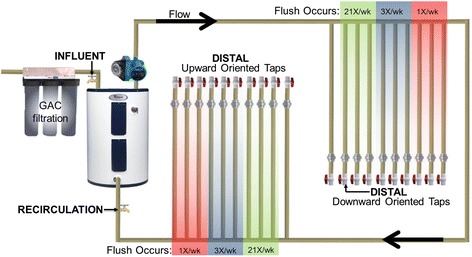
Overview of experimental design of replicated building plumbing systems. Two identical systems were constructed to examine the effect of water heater temperature setting and water use frequency on *Legionella* proliferation. One remained at 39 °C (control system) while the other was incrementally increased to 58 °C (experimental system) over 15 months. Influent water was flushed through three granular activated carbon whole-house filters (sample port Inf), a recirculating pump continuously pumped water around the return loop back to the water heater creating a completely mixed reservoir (sample port Recirc), and six replicate distal taps (three upward + three downward) were flushed at 3.8 L/min (1 gallon/min) 21 times/week, 3 times/week, and 1 times/week for a total of 36 pipes (sample ports: at end of distal pipes)

A major limitation of field studies is the inherent complexity encountered in actual building systems, which make it difficult to pinpoint precise factors that trigger *Legionella* proliferation. Therefore, our objective was to conduct a controlled, replicated laboratory investigation examining the interrelationship of water heater temperature set point and distal tap use frequency on *Legionella* occurrence. Identical experimental and control hot water plumbing systems were constructed in which continuously recirculating pipe loops delivered water to distal taps subject to high, medium, and low water use frequency (Fig. 1). Both systems were initially acclimated for 5 months at 39 °C to establish a baseline with stable microbial communities before incrementally increasing the water heater temperature of the experimental system to 42, 48, 51, and 58 °C, while the control system was maintained at 39 °C for the duration of the 15-month experiment. Genetic markers of *Legionella* spp. (23S ribosomal RNA (rRNA) gene), *L. pneumophila* (macrophage infectivity potentiator (*mip*) gene), *Vermamoeba vermiformis* (18S rRNA gene; an important ecological host for *Legionella*), and total bacteria (16S rRNA gene) were tracked by quantitative polymerase chain reaction (q-PCR) to measure re-growth in the recirculating lines relative to the influent water and in distal taps relative to the recirculating lines (Fig. 1). Given the current trend towards “green” buildings that are intended to conserve both water (i.e., to decrease water use frequency, which increases corresponding stagnation) and energy (i.e., lower temperature settings), the present moment is critical for untangling the complexities that trigger *Legionella* growth and identifying practical solutions for their control that can be considered in building system design.

## Results and discussion

Both *Legionella* spp. and *L. pneumophila* naturally colonized both the experimental and control building plumbing systems and established a comparable baseline, which provided the unique opportunity to systematically examine the effect of changes in building plumbing operation and microbial response under replicated and controlled conditions. Our overarching hypothesis was that *L. pneumophila* levels at the tap depend on the interrelationship between water heater temperature set point and use frequency and their collective influence on the microbiome. Table 1 breaks this hypothesis down more specifically, summarizing four representative conditions (I–IV) under which increased use frequency would be expected to increase, decrease, or have no effect on *L. pneumophila* levels. Across this study, we conducted testing with little to no disinfectant residual, as can occur in building plumbing, especially under water conservation scenarios and at the end of water main networks [29–32]. If disinfectant can be effectively delivered and maintained above about 0.5 mg/L as Cl_2_ (condition IV), it is generally believed that *L. pneumophila* will effectively be controlled [18, 22]. In the following sections, we first discuss physicochemical trends in temperature and chlorine and subsequently examine occurrence of *L. pneumophila* and other ecologically relevant microbes relative to these trends and in the context of the specific hypotheses presented in Table 1. Table 2 provides an overview of the calculations we employed in this study to compare the distribution of *L. pneumophila* between the experimental and control rigs and across various system compartments.

**Table 1:** Hypothesized effects of increased water use frequency under various hot water system operating conditions on *L. pneumophila* in distal taps

Condition^a^	Dominant impact	Hypothesized result	Experiment herein
I. No disinfectant and low water heater set point (*T* < 48 °C)	Growth due to increased delivery of nutrients to distal taps at ideal growth *T*	Greatest total numbers produced in distal taps with time but lower concentrations due to more frequent use	Control system, over time (*T* = 39 °C)
II. No disinfectant and moderate water heater set point (*T* = 48–51 °C)	Low-use condition provides optimal ecological selection by transient sub-lethal *T* events	Lower numbers produced in distal taps and lower concentrations at higher use	Exp. 2 (*T* = 51 °C)
III. No disinfectant and high water heater set point (*T* > 55 °C)	Re-growth limited only to distal taps during stagnation events	Lower concentrations	Exp. 3 (*T* = 58 °C)
IV. Stable and high disinfectant	Disinfection effect dominates	Lower number and concentrations	Not tested in this work

^a^See Fig. 2 for hypothetical temperature effects from the literature

**Table 2:** Calculations for determining *L. pneumophila* distribution across various system compartments and effects of operating conditions

Analysis	Calculation	Practical meaning/interpretation
Temperature effect on total planktonic loads (Table 3)	$\frac{{\left({\displaystyle \sum }{\left[\mathrm{L}\mathrm{p}\right]}_{\mathrm{Distal}}\times {\forall}_{\mathrm{Distal}}+{\left[\mathrm{L}\mathrm{p}\right]}_{\mathrm{Recirc}}\times {\forall}_{\mathrm{Recirc}}\right)}_{\mathrm{Control}}}{{\left({\displaystyle \sum }{\left[\mathrm{L}\mathrm{p}\right]}_{\mathrm{Distal}}\times {\forall}_{\mathrm{Distal}}+{\left[\mathrm{L}\mathrm{p}\right]}_{\mathrm{Recirc}}\times {\forall}_{\mathrm{Recirc}}\right)}_{\mathrm{Experimental}}}$	Factor by which planktonic *L. pneumophila* increase in the total system if the temperature is not elevated to the experimental setting
Temperature effect on the recirculating line and tank loads (Table 4)	$\frac{{\left({\displaystyle \sum }{\left[\mathrm{L}\mathrm{p}\right]}_{\mathrm{Recirc}}\times {\forall}_{\mathrm{Recirc}}\right)}_{\mathrm{Control}}}{{\left({\displaystyle \sum }{\left[\mathrm{L}\mathrm{p}\right]}_{\mathrm{Recirc}}\times {\forall}_{\mathrm{Recirc}}\right)}_{\mathrm{Experimental}}}$	Factor by which planktonic *L. pneumophila* increase in the recirculating portions of the system if the temperature is not elevated to the experimental setting
Temperature effect on distal tap loads (Table 3)	$\frac{{\left({\displaystyle \sum }{\left[\mathrm{L}\mathrm{p}\right]}_{\mathrm{Distal}}\times {\forall}_{\mathrm{Distal}}\right)}_{\mathrm{Control}}}{{\left({\displaystyle \sum }{\left[\mathrm{L}\mathrm{p}\right]}_{\mathrm{Distal}}\times {\forall}_{\mathrm{Distal}}\right)}_{\mathrm{Experimental}}}$	Factor by which planktonic *L. pneumophila* increase at the tap if the temperature is not elevated to the experimental setting
Re-growth in the recirculating lines (Fig. 3)	[Lp]_Recirc_ − [Lp]_Inf_	Re-growth in the recirculating lines relative to the influent
Concentration (Fig. 4a)	[Lp]	*L. pneumophila* concentration
Factor increase (Fig. 4b)	$\frac{{\left[\mathrm{L}\mathrm{p}\right]}_{\mathrm{Distal}}}{{\left[\mathrm{L}\mathrm{p}\right]}_{\mathrm{Recirc}}}$	Re-growth in the distal taps relative to the recirculating line
Weekly yield (Fig. 4c)	[Lp]_Distal_ × ∀ _Distal_ × Use	Total amount of *L. pneumophila* delivered at the tap per week
Biofilm re-growth (Fig. 5)	*L. pneumophila* in gene copies/cm^2^	Biofilm densities of *L. pneumophila*; because the same areas were re-swabbed, any *L. pneumophila* detected is assumed to have re-established during that experimental condition

*Lp*: *L. pneumophila* concentration in gene copies/mL, *Distal* distal tap, *Recirc* recirculating line and tank, *∀* volume, *Inf* influent, *Use* use per week (21, 3, or 1)

### Physicochemical trends

#### Distal pipe temperatures

**Fig. 2:**
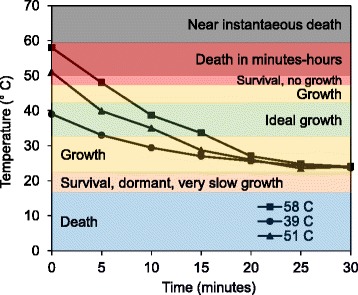
Water temperatures (and reported effects on *Legionella*) at distal taps with stagnation time. Targeted water temperatures were not maintained in pipes for sufficient durations after each use to effectively disinfect *Legionella. Shaded temperature regions* labeled on the plot represent the time required to achieve 90 % inactivation of *Legionella*. (time to 90 % death and growth temperature ranges based on references [42–48])

We documented a clear disconnect between water heater set point and temperatures observed at the distal taps. Water at the distal taps cooled to room temperature (26.1 ± 0.2 °C) within 25 min of each water use event, regardless of water heater set point temperature (Fig. 2). In general, water in the distal taps never exceeded the temperature × time requirements to achieve 99 % disinfection of *Legionella*. Stagnation temperature differed by only ~1 °C (Additional file 1: Figure S2), and *Legionella* spp. and *L. pneumophila* levels were not significantly different in upward versus downward oriented pipes (paired *t* test, *n* = 177, *p* value = 0.48 and 0.31, respectively), so these data were pooled for subsequent analysis, resulting in six replicates for each water use frequency.

#### Total chlorine

Chloramine was removed from influent Blacksburg, VA, drinking water using three granular activated carbon filters (Pentek, Upper Saddle River, NJ). Average total chlorine concentrations in the influent water samples were always less than 0.10 mg/L as Cl_2_ and remained near the detection limit (0.02 mg/L as Cl_2_) in the water heaters throughout the experiment (Additional file 1: Figure S3). Therefore, we achieved the goal of eliminating disinfectant from the system, which we hypothesize would have overridden the effects of temperature and water use that are the focus of this study (Table 1).

### General trends in *L. pneumophila* occurrence and effect of water heater temperature

**Fig. 3:**
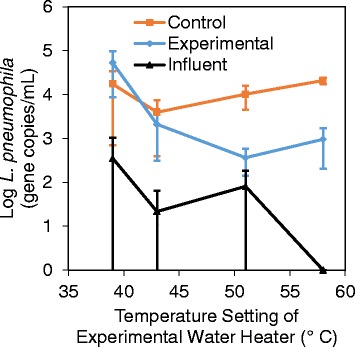
*L. pneumophila* concentrations in the recirculating lines compared to the influent. *L. pneumophila* concentration in the recirculating lines compared to the influent. The *x*-axis reports the temperature setting for the experimental water heater, with the corresponding values for the control and influent plotted for the same time point. The control system remained at 39 °C throughout the experiment. *Error bars* indicate 95 % confidence intervals on biological replicate samples (*n* = 2–6)

*L. pneumophila* was found to naturally colonize the systems at comparable levels following the 5-month baseline conditioning at 39 °C (Table 3), which facilitated subsequent comparisons throughout the study. Further, elevated levels of *L. pneumophila* in the recirculating lines relative to the influent across all samplings confirmed that at least some portion of the *L. pneumophila* detected was actively re-growing in the building plumbing and not just passing through from the influent water (Fig. 3, 1.7–3.5 logs higher in the recirculating lines; Kruskal-Wallis test, *p* value = 0.002–0.035, except the control system baseline sampling, *p* value = 0.11, and the experimental system at 51 °C, *p* value = 0.080). Unless otherwise stated, we focus our discussion here on the behavior of planktonic *L. pneumophila*, which is ultimately what consumers will be exposed to in buildings, and later describe what was observed with respect to other target microbes and in the biofilm.

**Table 3:** Average total number of planktonic *L. pneumophila* gene copies in each reservoir during each sampling (for each sampling, *n* = 18 for distal taps; *n* = 2–6 for tank + recirc)

		Baseline	Exp. 1	Exp. 2	Exp. 3
System	Reservoir	(5 months)	(8 months)	(13 months)	(15 months)
Control (always 39 °C)	Distal taps (water)	5.01E + 07	3.02E + 07	1.02E + 08	2.17E + 08
	Tank + recirc (water)	1.30E + 09	2.94E + 08	7.55E + 08	1.55E + 09
Experimental	Distal taps (water)	5.60E + 07	5.92E + 06	2.44E + 07	4.98E + 06
	Tank + recirc (water)	3.94E + 09	1.55E + 08	2.63E + 07	7.12E + 07
Control normalized to experimental		39 °C/39 °C	39 °C/42 °C	39 °C/51 °C	39 °C/58 °C
Total system *L. pneumophila* genes (water)		0.30	2.0	16.9	23.2
Tank + recirc *L. pneumophila* genes (water)		0.33	1.9	28.7	21.8
Distal tap *L. pneumophila* genes (water)		0.89	5.1	4.2	43.6

Generally, it was found that *L. pneumophila* decreased as the water heater temperature setting increased, as was apparent in comparing levels in the control versus experimental recirculating lines (Fig. 3). More detailed comparisons were made by normalizing the levels of *L. pneumophila* gene copies in the control to the experimental system as an indicator of how much higher they would be without the elevated temperature intervention (Table 3). When the experimental system was set to 51 °C, *L. pneumophila* was 28.7 times lower in the recirculating portion of the experimental system than the control system (Kruskal-Wallis test, *p* value = 0.019, *n* = 12), but the benefits of increased temperature were not observed at the distal taps until the highest experimental temperature setting, where *L. pneumophila* was 43.6 times lower in distal taps in the experimental system set to 58 °C than in the control system set to 39 °C (Kruskal-Wallis test, *p* value = 0.0005, *n* = 18) (Table 3). The overall trend illustrated that the elevated water heater temperature settings were more immediately effective in the recirculating lines, which are continuously exposed to the hot water, whereas higher temperature settings were needed to best control *L. pneumophila* at the tap, where the water stagnates and quickly cools.

#### *L. pneumophila* in the control system and effect of use frequency (condition I)

**Fig. 4:**
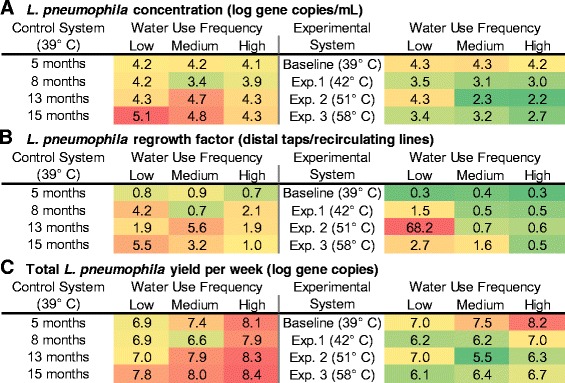
Heat map of *L. pneumophila* occurrence at the distal taps. Heat maps of *L. pneumophila* comparing **a** concentration in bulk water at each distal tap (log gene copies/mL), **b** distal taps normalized to the recirculating lines (re-growth factor), and **c** total yield of *L. pneumophila* per week at the tap (log gene copies). *Colors* are on a continuous scale from *green* (low) to *red* (high). Table 3 provides a detailed description of each calculation

Examination of the control system provided the opportunity to directly evaluate the effect of water use frequency, as described in condition I (Table 1). Interestingly, we observed that there was initially little difference in the concentration of *L. pneumophila* (gene copies/mL) as water use frequency changed (Fig. 4a; Kruskal-Wallis test, *p* value = 0.31–0.52). However, this initial assessment can be deceiving as the actual yield of *L. pneumophila* at the tap (gene copies per week) typically increased by about 1 log from low use to high use because the concentrations are multiplied by the number of times per week each tap was used (Table 2; Fig. 4c). This trend was also true for the experimental system when operated at the baseline condition before the temperature was elevated. We hypothesize that this phenomenon is due to increased delivery of nutrients in the recirculating line, which broadly stimulates the microbial community in the water delivered to the distal taps. If true, this would suggest that increasing water use frequency alone will not necessarily fix a *Legionella* problem associated with stagnant conditions and could partially explain discrepancies in the effects of stagnation in prior reports [19, 23–25].

Comparing the distal taps to the recirculating lines is another approach to evaluate the effect of use frequency and stagnation (Fig. 4b). The *L. pneumophila* re-growth factor (defined in Table 2) under condition I tended to strengthen with time, indicating that *L. pneumophila* could become more concentrated under the stagnant conditions at distal taps relative to the recirculating line as a system ages. Specifically, the *L. pneumophila* growth factor was less than 1 for all three water use conditions at the time of the baseline sampling but increased to 5.5 and 3.2 in the low- and medium-use frequencies, respectively, by 15 months (Fig. 4b).

Monitoring the control system with time was also essential for this study in order to be certain that the trends observed in the experimental condition were a result of the temperature elevation and not necessarily natural succession of the microbial populations. Notably, *L. pneumophila* levels generally increased with time at the tap of the control system over the 15-month study (Table 3, by a factor of 4.3; Kruskal-Wallis test, *p* value <0.0001, *n* = 16–18 per sampling event), especially in the low-use condition (Fig. 4a). By the end of the study, *L. pneumophila* was 6.3 times higher (1.1 × 10^5^ gene copies/mL) in the low-use relative to high-use distal taps (a factor of 6.3) (Kruskal-Wallis Test, *p* value = 0.004), suggesting that differences induced by water use frequency became more pronounced as the microbial ecology of the systems matured. In contrast, *L. pneumophila* levels were relatively stable with time in the recirculating portions of the system (Table 3; Fig. 3, Kruskal-Wallis test, *p* value = 0.22–0.40; *n* = 6 per sampling event). Consistent with the nutrient delivery hypothesis, this suggests that a stable microbial ecology may take longer to establish at the tap, where flow is intermittent, than in a continuously flowing system. A random survey of 452 household hot water systems also suggests that it may take time for *Legionella* to colonize new pipes, where it was found that homes with new plumbing systems (<2 years old) had no *Legionella* spp.-positive samples while 14 % of older homes were colonized [25].

#### *L. pneumophila* in the experimental system at moderate temperature (51 °C) (condition II)

A major finding of this study may best be described as an ecological “sweet spot” that occurred when the water heater was set at 51 °C and the water use frequency was low. In this specific condition, enrichment of *L. pneumophila* at the tap relative to the recirculating line was striking (68.2 times higher; Fig. 4b). Interestingly, *L. pneumophila* concentrations decreased at the tap as expected in the medium- and high-use scenarios relative to both low use and the recirculating lines as the temperature was elevated to 51 °C (Fig. 4a, b), suggesting a unique phenomenon when a moderate water heater temperature is combined with low water use frequency. Besides being enriched relative to the recirculating line, *L. pneumophila* under the 51 °C/low-use condition was also uncharacteristically high in concentration (Fig. 4a), equivalent to that of the control system maintained at optimal growth temperature (Kruskal-Wallis test, *p* value = 1.0), and was the only case where low-use distal taps yielded greater total *L. pneumophila* than high-use distal taps (Fig. 4c, by a factor of 5, Kruskal-Wallis test, *p* value = 0.044). We hypothesize that a brief exposure to a sub-optimal disinfection temperature (i.e., Fig. 2) combined with sufficient stagnation time for recovery and re-growth can lead to selection of *L. pneumophila* at the tap. Others have also noted evidence that brief exposures to elevated temperatures could have unintended negative consequences by decreasing competition or enhancing nutrient availability via necrotrophic growth [33, 34], and rapid recolonization after thermal disinfection has been observed in the field [35]. Importantly, new guidelines on effective control of *Legionella* in building systems suggest maintaining at least 51 °C in all portions of the hot water system [18, 22]. It is apparent from these results that it will be difficult (if not impossible) to maintain set point temperatures throughout distal portions of the system (Fig. 2; Additional file 1: Figure S2) and may inadvertently increase *Legionella* risk under certain circumstances. The 51 °C sweet spot warrants further investigation.

#### *L. pneumophila* in the experimental system at high temperature (58 °C) (condition III)

While elevating the water heater temperature to 58 °C effectively eliminated the selective effect of the 51 °C/low-use condition, the advantages were not striking in terms of *L. pneumophila* concentrations (Fig. 4a) or yields (Fig. 4c) in medium- or high-use distal taps relative to 42 or 51 °C. Nevertheless, the advantages of elevated water heater temperature were clear when comparing the experimental to the control system (Fig. 4; 40–50 times reduction in total weekly yield at 58 versus 39 °C), suggesting that the gradual *L. pneumophila* colonization of both systems with time may have muted the benefits of the elevated temperature. Further, *L. pneumophila* tended to be positively selected at the tap in the control system (Fig. 4b, ratios generally >1.0) and negatively selected at the tap in the experimental system at 58 °C/high-use frequency (Fig. 4b, ratios <1.0). This suggests that, if applied properly, elevated temperature can have a lasting effect for *L. pneumophila* control at the tap. Interestingly, the enhanced delivery hypothesis appeared to hold true as the temperature was elevated in the experimental system, with increased total yields of *L. pneumophila* as water use frequency increased (Fig. 4c). However, increased water use decreased *L. pneumophila* concentrations by a factor of 5.0 relative to lower use in the experimental system at 58 °C (Fig. 4a).

### Microbial ecological relationships of *L. pneumophila* in hot water plumbing

#### Trends in biofilm-associated *L. pneumophila*

**Fig. 5:**
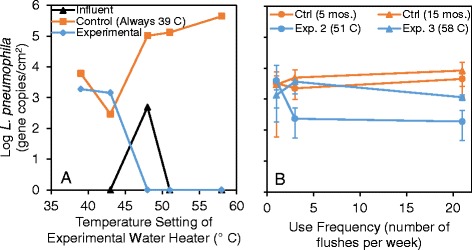
Biofilm-associated *L. pneumophila* concentrations. **a** *L. pneumophila* concentrations in recirculating lines as a function of water heater temperature setting. The *x*-axis reports the temperature setting for the experimental water heater, with the corresponding values for the control and influent plotted for the same time point. The control system remained at 39 °C throughout the experiment. No error bars were calculated due to the biofilm sampling approach used. **b** *L. pneumophila* concentrations at the distal taps as a function of flush frequency. *Error bars* indicate 95 % confidence intervals on biological replicate samples (*n* = 6). Note that biofilms were subject to repeated sampling of the same area; thus, the numbers represent re-growth between sampling events

We repeatedly swabbed the same area (65 cm^2^) to collect biofilm at the end of each experimental period, providing a measurement of *L. pneumophila* that re-colonized pipe surfaces at each temperature setting (Fig. 5). *L. pneumophila* in influent pipe biofilms was consistently below the detection limit, except for the 11-month sampling date (Fig. 5a, during a period of elevated influent water temperature of 22–23 versus 11–13 °C for subsequent sampling events), further lending confidence that *L. pneumophila* gene copies observed in the plumbing systems were representative of re-growth and not an artifact of the influent. Interestingly, *L. pneumophila* levels were consistently below detection in the recirculating pipe biofilm of the experimental system when the water heater setting was ≥48 °C, while they consistently increased with time in the control rig set to 39 °C (Fig. 5a). Thus, it appears that *L. pneumophila* was not adept at re-colonizing biofilms at moderate-high water heater temperature set points, though it cannot be certain how it behaved in intact portions of the biofilm not subject to re-sampling.

Water use frequency also appeared to affect re-growth of biofilm-associated *L. pneumophila*. For example, in the control system biofilm, *L. pneumophila* increased with increased use frequency, with 55 times more *L. pneumophila* in the continuously recirculating line than the most frequently used distal taps by the end of the study (Fig. 5a versus 5b). This is consistent with the nutrient delivery hypothesis [23]. However, where there was a trend in the experimental system, it was the opposite, with 19.2 times less biofilm-associated *L. pneumophila* in high-use distal taps than low-use taps when the heater was set at 51 °C (Fig. 5b, Kruskal-Wallis test, *p* value = 0.037, *n* = 12). Notably, this was also the ecological sweet spot condition noted above, suggesting the brief exposure to sub-optimal disinfection temperature followed by long stagnation selected for *L. pneumophila* in the biofilm as well as the bulk water. Although water use frequency can be subordinate to other factors, such as temperature and corresponding microbial ecological responses, analyzing water use conditions in conjunction with water temperature helps reconcile discrepancies in prior reports of effects of stagnation on *L. pneumophila* [15, 23, 25].

#### Relationships among *L. pneumophila* and other ecologically relevant microorganisms

**Fig. 6:**
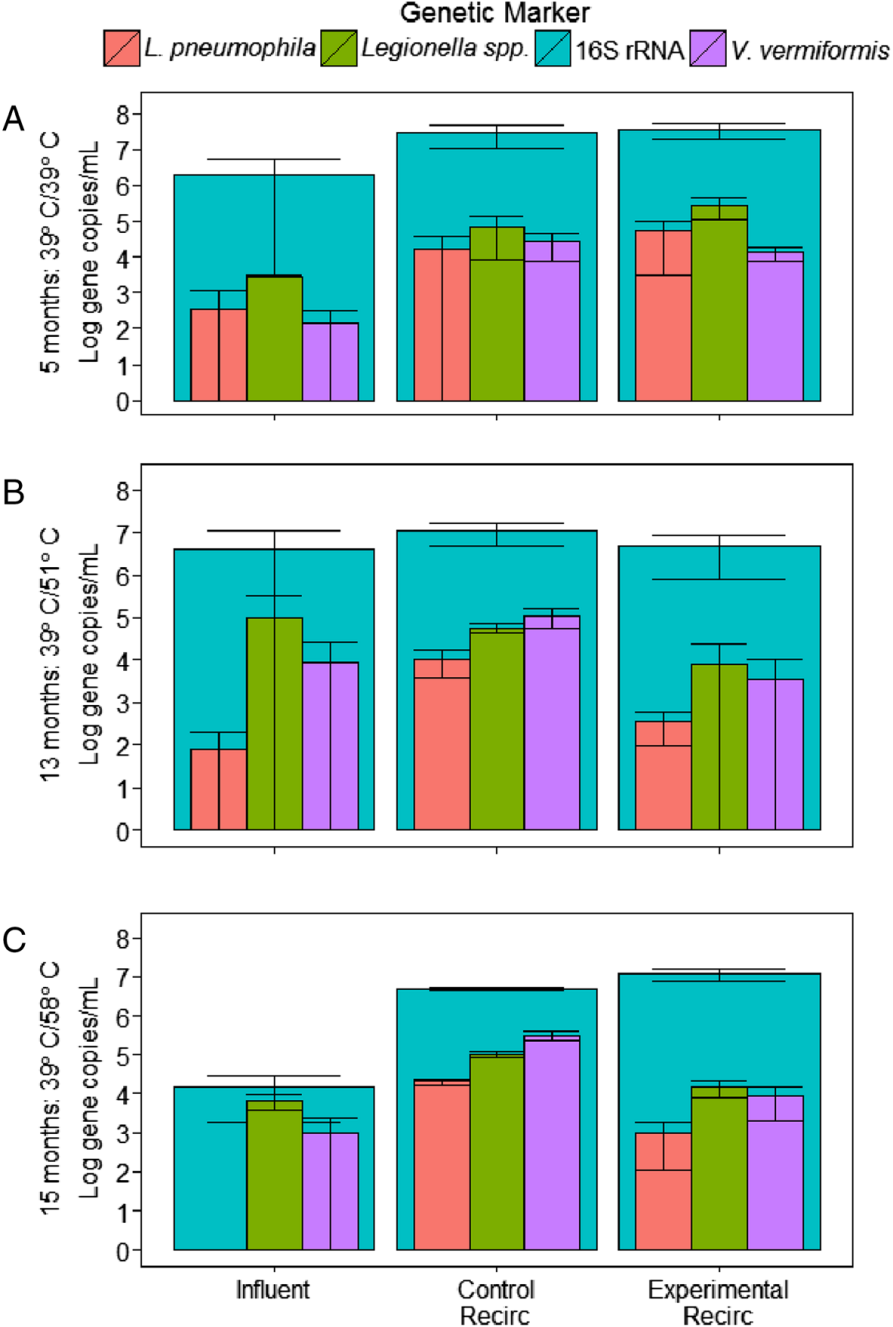
Relative levels of *L. pneumophila* and ecologically relevant microbes in the influent and recirculating line. Log *L. pneumophila*, *Legionella* spp., and *V. vermiformis* nested within total bacteria concentrations (gene copies/mL) in the influent and recirculating lines for **a** the baseline sampling (both systems set to 39 °C at 5 months), **b** exp. 2 (control system set to 39 °C, experimental system set to 51 °C at 13 months), and **c** exp. 3 (control system set to 39 °C, experimental system set to 58 °C at 15 months)

**Fig. 7:**
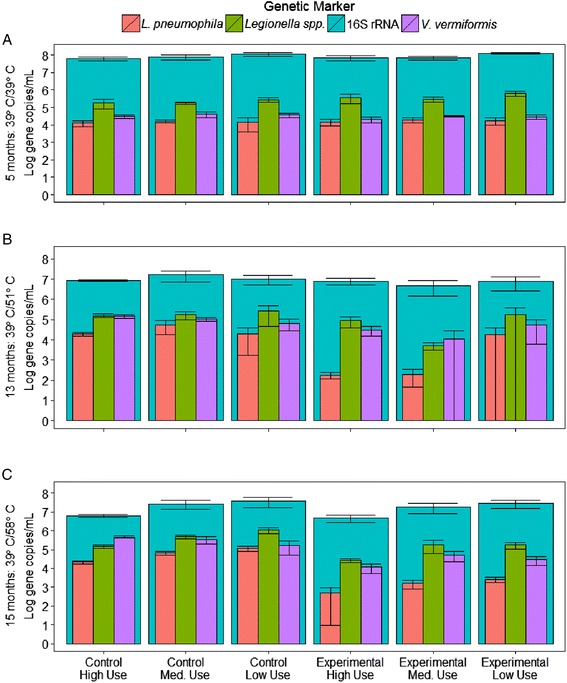
Relative levels of *L. pneumophila* and ecologically relevant microbes in the distal taps. Log *L. pneumophila*, *Legionella* spp., and *V. vermiformis* nested within total bacteria concentrations (gene copies/mL) in the distal taps for each water use frequency for **a** the baseline sampling (both systems set to 39 °C at 5 months), **b** exp. 2 (control system set to 39 °C, experimental system set to 51 °C at 13 months), and **c** exp. 3 (control system set to 39 °C, experimental system set to 58 °C at 15 months)

Relationships were explored among total bacteria, *Legionella* spp., and *V. vermiformis* to gain insight into how *L. pneumophila* behaved in the context of the broader plumbing microbiome (Figs. 6 and 7). Remarkably, elevated temperatures did not have a significant effect on the levels of total bacteria in the recirculating lines or at the tap (Kruskal-Wallis test, *p* value = 0.27, *n* = 58). While it was expected that the disinfecting properties of the hotter water would reduce total microbial populations, our results suggest that instead the elevated temperature merely shifted the microbiome composition, which can be seen by reductions in the other specific targets in the experimental relative to the control system (Figs. 6 and 7).

Of particular interest was the relationship between *Legionella* and *V. vermiformis*, which is among free-living amoebae thought to act as obligate hosts for *Legionella* replication in drinking water systems and thus could be an important player in pathogen control [27, 36, 37]. While there is a broad range of known amoeba hosts for *Legionella*, *V. vermiformis* was chosen as the focus of this work because it is among the most frequently detected *Legionella* host organisms in drinking water [38–40] and was found to be the most prevalent amoebae (and weakly correlated to *Legionella* spp.) in a prior investigation of Blacksburg, VA, tap water [36]. Here, we found that *Legionella* spp. and *L. pneumophila* were correlated with *V. vermiformis* under certain circumstances. During the baseline sampling, when the microbial community was still developing, there were no correlations between *V. vermiformis* and *Legionella* spp. or *L. pneumophila* (Spearman rank correlations, rho = −0.19–0.47, *p* value = 0.15–0.47). However, later in the experiment (13 months), significant correlations developed in the distal pipes in the mature experimental system set to 51 °C (Spearman rank correlation, rho = 0.52–0.68, *p* value = 0.002–0.031). This suggests that *V. vermiformis* may have played a role in the much higher levels of *L. pneumophila* observed in the water- and biofilm-associated *L. pneumophila* as thermal stresses reached the sweet spot in the experimental system at 51 °C (Fig. 4b, c). While correlations did occasionally exist in the recirculating line samples, seven of eight correlations of *V. vermiformis* compared to *Legionella* spp. and *L. pneumophila* during the last two sampling periods were inconsistent and insignificant (Spearman rank correlation, rho = 0.02–0.70, *p* value = 0.19–0.95). Lack of a consistent correlation suggests a dynamic relationship between *V. vermiformis* and *Legionella*, which is intuitive given their predator-prey relationship.

#### Relationship between *Legionella* spp. and *L. pneumophila*

The genus *Legionella* contains other pathogens, besides *L. pneumophila*, as well as non-pathogenic members. Thus, there is interest in how *L. pneumophila* behaves in hot water systems relative to *Legionella* spp. *L. pneumophila* and *Legionella* spp. were strongly correlated across all water samples (*R*^2^ = 0.70, *n* = 484) and across all distal tap water samples (*R*^2^ = 0.75, *n* = 357), but in most other cases, correlations were weak (e.g., *R*^2^ = 0.57, n=90) in water samples of recirculating lines or non-existent. This indicates that there are situations under which *L. pneumophila* trends with other *Legionella* spp. and other cases where it does not. In particular, we observe an apparent decrease in the ratio of *L. pneumophila* to *Legionella* spp. with elevated temperature. For instance, when the temperature of the experimental system was increased to 48 or 58 °C (but not 51 °C possibly due to the unique selective condition), the ratio of *L. pneumophila* to *Legionella* spp. was significantly lower in the experimental than in the control system (paired *t* test, *p* value <0.0001, *n* = 36–48). While temperature may truly be the dominating factor influencing the type of *Legionella* that prevails, other selectors have been noted in the literature, such as other microorganisms (e.g., *Bacillus subtilis*) inhibiting *L. pneumophila* growth within amoeba or lysing cells [34, 41, 42].

#### Survival of *L. pneumophila* at elevated temperatures

Importantly, this study demonstrated that, even at the highest temperature of 58 °C, *L. pneumophila* was not eliminated from the hot water plumbing and continued to persist at levels greater than the influent (Fig. 3). We did not expect this result given that it is thought that *L. pneumophila* is unable to replicate above 50 °C [49–53], though it has been observed to survive short periods of time at 55–70 °C and long periods (on the order of months) as free organism in hot spring water [43–51]. Nonetheless, our work is strongly suggestive that *L. pneumophila* growth does occur in this temperature range under representative plumbing conditions. Given that 99.97 % (3 logs) of planktonic *L. pneumophila* would theoretically be washed out of both systems each week, re-growth is the most likely explanation for the persistence observed at elevated temperature. Even though biofilm-associated *L. pneumophila* was shown to not be able to re-colonize the swabbed areas at higher temperatures, it is possible that *L. pneumophila* persisted in and was released from the vast majority of the biofilm not disturbed by sampling, perhaps within amoebae hosts. Notably, high levels of planktonic *V. vermiformis* was detected at 58 °C (Fig. 6, average of 8.4 × 10^3^ gene copies/mL), which could extend the range at which *L. pneumophila* grows [14, 52].

## Conclusions

Here, we examined the effect of water heater temperature setting and water use frequency, which are two critical factors for energy and water conservation, on *L. pneumophila* as a representative OP resident to the building plumbing microbiome. This controlled, replicated, pilot-scale approach aided in resolving complexities encountered in prior field studies and addressing discrepancies with respect to effects of temperature and stagnation reported in the literature. Overall, it was found that elevated temperature was a critical factor in suppressing *L. pneumophila* growth both in continuously recirculating hot water lines and at the tap, where water quickly cools to room temperature following heat shock. Nonetheless, naturally occurring *L. pneumophila* persisted up to 58 °C, with strong evidence for growth within this pilot-scale plumbing system, relative to prior understanding that it does not grow above 50 °C under simplified laboratory conditions. Further, it was found that temperature and water use frequency can have interactive effects; for example, optimal *L. pneumophila* selection at the tap was observed when the water use frequency was low following a heat shock at 51 °C. At the same time, while higher use frequency can dilute *L. pneumophila* and result in lower concentrations at the tap, it still tended to result in higher overall yields, given that concentration is multiplied by higher use frequency. We hypothesize that increased water use frequency replenishes nutrients required for *L. pneumophila* re-growth and delivers a measurable *L. pneumophila *population to distal taps, where temperature conditions remain suitable for *L. pneumophila* re-growth or persistence. Overall, this study takes a step towards untangling the complexity of the factors shaping the microbial ecology of hot water plumbing and lays the groundwork for an integrated approach for opportunistic pathogen control.

## Methods

### Experimental setup and operation

Two identical household hot water systems with 71.9-L (19 gallons) electric water heaters and continuously recirculating pipe loops were constructed with nominal ¾-in. chlorinated polyvinyl chloride (CPVC; Charlotte Pipe, Charlotte, NC) pipe (Fig. 1). Each system tested two pipe orientations (upward/downward) with three water use patterns in triplicate, including low use (1 flush/week), medium use (3 flushes/week), and high use (21 flushes/week) for a total of 36 distal taps (2 systems × 2 orientations × 3 use patterns × triplicate = 36). Each distal tap pipe was 1.7 m (5.5 ft) for a total distal tap volume of 0.43 L (0.11 gallons) and internal surface area of 0.87 m^2^ (0.94 ft^2^). Each recirculating line was a total of 7.6 m (25 ft). The water heaters and recirculating lines were completely mixed, resulting in a combined volume of 73.9 L (19.5 gallons) and surface area of 1.46 m^2^ (15.7 ft^2^). Each flush was conducted for 28 s at 3.8 L/min (1 gallon/min). Influent water consisted of well-flushed (10 min at 11.3 L/min), granular activated carbon (GAC)-filtered Blacksburg, VA, tap water. Both systems were initially acclimated for 5 months at 39 °C. Afterwards, the experimental system water heater temperature was increased approximately by 5 °C increments while the control system remained at 39 °C. During periods of stagnation, distal pipes cooled to room temperature.

### Water quality analysis

Disinfectant residual, total ammonia, temperature, pH, dissolved oxygen (DO), total organic carbon (TOC), and total and dissolved cations were generally characterized at each temperature setting beginning 1 week after each temperature adjustment. Chloramine and total ammonia were measured according to Standard Method 4500-Cl25 and 5310-NH3 using a DR2700 or DR5000 spectrophotometer (HACH, Loveland, CO). pH and temperature were measured using a pH 110 meter with automatic temperature correction (Oakton Research, Vernon Hills, IL). DO was monitored using a Thermo Scientific Orion 3-star meter. TOC was measured by persulfate-ultraviolet detection using a Sievers Model 5300C with an autosampler according to Standard Method 5310 C. Cations were measured by inductively coupled plasma mass spectrometry after acidification with 2 % nitric acid (*v*/*v*) and >24-h holding time.

### Microbiological sample collection and DNA extraction

After a minimum of 2-month acclimation period at each experimental condition, approximately 0.5 L of first-flush water was collected directly from the influent, recirculating lines, and each distal tap at the end of regular stagnation periods for each use condition and filtered through sterile 0.22-μm pore-size mixed cellulose ester filters (Millipore, Billerica, MA). Filters were fragmented and subjected to DNA extraction. For biofilm sampling, 65 cm^2^ of the influent, recirculating line, and ends of the distal tap pipes accessible by threaded union connections were swabbed using sterile cotton-tip applicators (Fisherbrand, Fisher Scientific, UK). DNA was extracted directly from the fragmented filters and cotton swabs using a FastDNA Spin Kit (MP Biomedicals, Solon, OH) according to the manufacturer protocol. Field, trip, and equipment negative controls consisting of pre-sterilized water in identical sampling bottles were included each time samples were collected.

### Quantitative polymerase chain reaction

Gene markers for *Legionella* spp., *L. pneumophila*, and *V. vermiformis*, along with bacterial 16S rRNA genes, were enumerated by quantitative polymerase chain reaction (q-PCR) assays using previously established methods [42]. In brief, all q-PCR assays were performed in 10 μL reaction mixtures containing SsoFast Probes or Evagreen Supermix (Bio-Rad, Hercules, CA), 250 or 400 nM primer, and 93.75 nM probe (Taqman assay only) with 1 μL of DNA template. DNA extracts (diluted 1:10 to minimize inhibition), a negative control, 10-fold serial dilutions of standards, and a positive spike into sample DNA matrix were included in triplicate wells with each q-PCR run. The quantification limit (QL) for all q-PCR assays ranged from 10 to 1000 gene copies/reaction and was implemented as appropriate for each run. Samples yielding threshold cycles ≥ QL in at least two q-PCR triplicate wells were considered quantifiable. Samples with only one triplicate above the QL threshold cycle or samples otherwise below the QL were re-analyzed undiluted to increase the QL of the assays. On each re-run plate, standard DNA template was spiked into the experimental DNA matrix to confirm that amplification reactions were not inhibited in undiluted samples. If inhibited, the sample was marked as below the QL. All values are reported as log(gene copies/mL + 1).

### Statistical analyses

All error bars on figures and ±calculations are 95 % confidence intervals, calculated based on the normal cumulative distribution function, degrees of freedom, and standard error. For graphing and statistical purposes, any positive detection below q-PCR QL was entered as half of the quantification limit. All data exploration was conducted in Microsoft Excel 2013 or JMP Pro 11. Spearman’s rank coefficient and associated significance tests were conducted in JMP Pro 11 to detect and quantify relationships between gene markers (using “Multivariate Methods”). Other statistical tests were performed using RStudio with R version 3.2.0. Student’s *t* test (“t.test()”) and Kruskal-Wallis tests with a Holm *p* value adjustment for multiple comparisons were conducted to compare sample means (initially with “kruskal.test(),” then using package and function “dunn.test()” for multiple comparisons). Significance was determined at *p* = 0.05.

## Additional files

Additional file 1:
**Tables S1–S3 and Figures S1–S8.** This contains Tables S1–S3 and Figures S1–S8 [45–59]. (DOCX 77.1 kb) Additional file 2:
**Correlation Analysis**. This contains all correlations analyses of quantitative polymerase chain reaction assays. (XLSX 24.2 kb)
