# Supplementary material for: Water heater temperature set point and water use patterns influence Legionella pneumophila and associated microorganisms at the tap
Source: Microbiome. 2015 Dec 1;3:67. doi: 10.1186/s40168-015-0134-1 (PMC4666224; doi:10.1186/s40168-015-0134-1)
Supplement: Additional file 1: — Tables S1–S3 and Figures S1–S8. This contains Tables S1–S3 and Figures S1–S8 [45–59]. (DOCX 77.1 kb) [file 40168_2015_134_MOESM1_ESM.docx]

Water heater temperature set point and water use patterns influence *Legionella pneumophila* and associated microorganisms at the tap

William J. Rhoads^*^, Pan Ji, Amy Pruden, Marc A. Edwards

Charles E. Via Department of Civil and Environmental Engineering, Virginia Tech, Blacksburg, VA 24061

^*^Corresponding Author. Email: [wrhoads@vt.edu](mailto:wrhoads@vt.edu), Phone: (417) 437-2550; Fax: (540) 231-7916

Supporting Information

**Table S1. P-values for Kruskal-Wallis multiple comparisons with Holm p-value adjustment for planktonic *L. pneumophila* concentrations (gene copies/mL) in the influent and recirculating lines (See Figure 3, n=2 for Influent baseline; otherwise n=6 for each system)**

| **Comparison** | **Baseline (39° C v 39° C)** | **Exp. 1 (39° C v 42° C)** | **Exp. 2 (39° C v 51° C)** | **Exp. 3 (39° C v 58° C)** |
| --- | --- | --- | --- | --- |
| **Influent vs Control** | **0.11** | **0.0019** | **0.0051** | **0.0002** |
| **Influent vs Experimental** | **0.016** | **0.0074** | **0.080** | **0.035** |
| **Control vs Experimental** | **0.084** | **0.29** | **0.019** | **0.033** |

**Table S2. P-values for Kruskal-Wallis multiple comparisons with Holm p-value adjustment for planktonic *L. pneumophila* concentrations (gene copies/mL) in distal taps as a function of flow frequency (See Figure 4a)**

| **Comparison** | **Control System, Baseline (5 mos,**  **n=16)** | **Control System, Sample 4 (15 mos, n=18)** | **Experimental System,**  **39° C (5 mos,**  **n=18)** | **Experimental System,**  **42° C (8 mos,**  **n=18)** | **Experimental System,**  **48° C (11 mos,**  **n=18)** | **Experimental System,**  **51° C (13 mos,**  **n=18)** | **Experimental System,**  **58° C (15 mos,**  **n=18)** |
| --- | --- | --- | --- | --- | --- | --- | --- |
| **High vs Medium** | **0.31** | **0.027** | **0.50** | **0.41** | **0.010** | **0.43** | **0.058** |
| **High vs Low** | **0.34** | **0.0004** | **0.52** | **0.088** | **0.18** | **0.0003** | **0.0015** |
| **Medium vs Low** | **0.43** | **0.080** | **0.37** | **0.094** | **0.088** | **0.042** | **0.080** |

**Table S3. P-values for Kruskal-Wallis with Holm p-value adjustment for multiple comparisons for total weekly planktonic *L. pneumophila* yield (gene copies) in distal taps as a function of flow frequency (See Figure 4c)**

| **Comparison** | **Control System (all samplings, n=90)** | **Experimental System,**  **39° C (5 mos,**  **n=18)** | **Experimental System,**  **42° C (8 mos,**  **n=18)** | **Experimental System,**  **48° C (11 mos,**  **n=18)** | **Experimental System,**  **51° C (13 mos,**  **n=18)** | **Experimental System,**  **58° C (15 mos,**  **n=18)** |
| --- | --- | --- | --- | --- | --- | --- |
| **High vs Medium** | **<0.0001** | **0.052** | **0.099** | **0.052** | **0.033** | **0.31** |
| **High vs Low** | **<0.0001** | **0.0001** | **0.084** | **0.0001** | **0.44** | **0.42** |
| **Low vs Medium** | **0.012** | **0.026** | **0.46** | **0.026** | **0.044** | **0.29** |


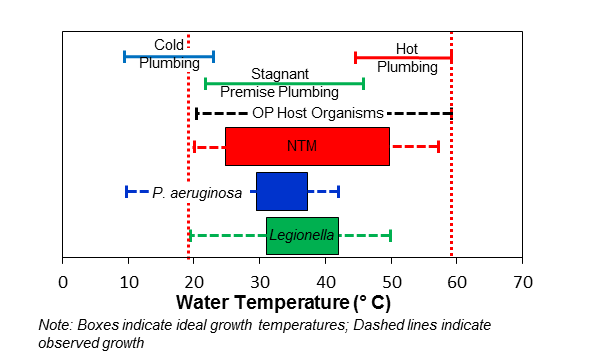


**Figure S1. Opportunistic pathogen growth and survival temperatures relative to premise plumbing water temperatures**

**Figure S1 Legend: Solid boxes indicate ideal growth temperatures associated with reference opportunistic pathogens (non-tuberculous mycobacteria (NTM), *Pseudomonas aeruginosa*, and *Legionella*) and amoeba host organisms (e.g., *Vermamoeba* and *Acanthamoeba*) of important to premise plumbing ecology, dashed lines indicate the temperature ranges in which growth and/or survival of each organism has been documented, and the solid lines indicate water temperatures commonly encountered in cold and hot water supplies as well as stagnant premise plumbing.[45-59]**

**Figure S2. Temperature measurements in the control and experimental system in upward and downward oriented pipes for all five experimental conditions; error bars represent 95% confidence intervals on the triplicate pipes.**

Upward oriented pipes were significantly warmer than downward oriented pipes (paired t-test, n=318, p-value<0.0001); however, the difference was small (on average 1.1° C). Generally speaking, water in the distal taps never exceeded the temperature and time requirements to achieve 99% disinfection of *Legionella*, and, as a result, here were also no significant differences in *L. pneumophila* or *Legionella* spp. genetic marker concentrations between upward and downward pipes (paired t-test, n=177, p-value = 0.31-0.48).

**Figure S3. Total chlorine concentrations in the influent and both recirculating lines throughout the study (detection limit = 0.02 mg/L as Cl_2_); error bars represent 95% confidence intervals on repeat sampling of the recirculating line at the beginning and end of stagnation periods for the distal taps (n=9-12 for each system at each sampling period).**

**Total Organic Carbon (TOC).** Organic carbon initially leached from the chlorinated polyvinyl chloride (C-PVC) pipes at about 3 mg/L during the first two weeks of startup operation. During the experimental phase, TOC increased marginally in the recirculating lines by an average of 0.07-0.08 mg/L compared to the influent. TOC initially increased in distal taps by 0.50-0.52 mg/L during the baseline testing, and over time TOC leaching was reduced to 0.17-0.21 mg/L during the last sampling period (Figure S4).

**Figure S4. Total organic carbon concentrations in the control and experimental system distal taps during each sampling period; error bars represent 95% confidence intervals on two samplings of the triplicate pipes and repeat independent samples of the recirculating lines .**

**Figure S5. Biofilm *L. pneumophila* concentration as a function of flush frequency per week in the control system**

**Figure S6. Biofilm *L. pneumophila* concentration as a function of flush frequency per week in the experimental system**


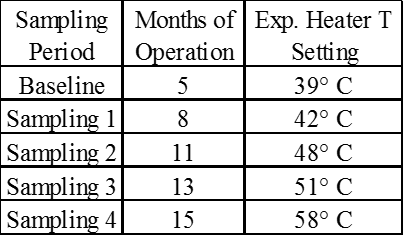


A

**Figure S8. Ratio of *L. pneumophila* to *Legionella* spp. in the distal tap pipes for A) the control system and B) the experimental system for each flush frequency across all samples; Error bars represent 95% confidence intervals on the six biological replicates for each condition.**
